# From Abstinence to Assistance: Antinatalism's Unexpected Endorsement of the Principle of Procreative Beneficence

**DOI:** 10.1111/bioe.13432

**Published:** 2025-06-11

**Authors:** Marcus T. L. Teo

**Affiliations:** ^1^ Centre for Biomedical Ethics, Yong Loo Lin School of Medicine National University of Singapore Singapore Singapore

**Keywords:** antinatalism, Principle of Procreative Beneficence, reproductive ethics

## Abstract

This essay begins from the point that developments in antinatalism, or the view that it is wrong to bear children, place legitimate pressures on prospective parents to seriously consider the harms of bringing their prospective children into existence. This essay does not defend antinatalism but instead considers an upshot of bioethical import if one takes these antinatalist pressures seriously. Attending to the debate on the normative legitimacy of Savulescu's Principle of Procreative Beneficence (PPB), I argue that antinatalist pressures give rise to reasons that count in favor of the PPB. I show how an antinatalist‐corollary version of the PPB might be derived and how we might respond to the PPB's main criticisms and conceptual difficulties.

Antinatalism is the normative view that one should not bear children. However, descriptively, it seems obvious that we (i.e., humanity) will continue to. Despite this, it is not yet time for antinatalism to make its exit from applied bioethical inquiry. This is because I think that antinatalism still has plenty to teach us about reproductive ethics. That is, even if we do not accept antinatalism, developments in antinatalist literature may shed new light on existing puzzles. In this work, I study the puzzle of the legitimacy of the Principle of Procreative Beneficence (PPB). The PPB, since its promulgation by Julian Savulescu [1], has been used as a springboard from which ethical upshots of practical import may be derived (e.g., in genetic engineering [2] and genome editing [3]). However, the PPB has been the subject of intense scholarly debate [4, 5, 6, 7, 8], and it seems like bioethicists remain divided as to whether the PPB holds legitimate value in telling us about our procreative duties. This study, therefore, intends to contribute to the literature by offering a novel point of support for the PPB via antinatalism. Specifically, I argue that the kinds of pressures that antinatalism places on parents, if one takes them seriously, provide support for the PPB.

To be clear, I do not aim to defend antinatalism in this paper, nor do I intend to offer an overview of antinatalist philosophy. I attend only to the observation that antinatalism has given rise to legitimate pressures that prospective parents have normative reasons to respond to in justifying their decision to procreate. It is important to note that these pressures do not necessarily arise from antinatalist advocacy; pronatalists also admit that these pressures are legitimate and that prospective parents ought to respond to them all the same. Len Doyal [9], for example, writes that potential parents ought to seriously consider the potential harms that may bring suffering to their prospective children. Pronatalist David Wasserman [10], as well, points out that although having children might be justified, we ought to strengthen justifications for having children despite the harms and risks of bringing them into the world. It suffices, then, for us to consider that without defending antinatalism per se, we might be apt to look into deriving meaningful normative upshots that arise from antinatalism. Relevant to this paper is the observation that coming into existence always entails some degree of harm, which is central to antinatalism. Note that this is distinct from Benatarian antinatalism, where Benatar argues that all lives are a net harm [11]. We need not accept a claim as bold as Benatar's for this study. The observation I point to is far more modest: that all existence entails some degree of risk or harm. For the purposes of this paper, then, I shall remain agnostic to whether prospective parents can reasonably justify procreation by outweighing, silencing, or simply negating the harms and risks that procreation entails. Further, an assumption that I should note is that parents are causally and morally responsible for the harms and risks that they impose upon their children. Of course, this is contestable, and the extent to which arguments are successful depends heavily on many factors outside of the remit of this paper. For example, a Confucian view might not accept what I assume in this paper [12]. Nonetheless, I carry on with the assumptions that I have laid out, with a second‐order assumption that they are intuitive. It should also be noted that I do not engage with the empirical, scientific details of how the PPB might be actionable. Specifically, I do not consider whether the present state of prenatal genetic diagnosis (PGD) makes possible the kinds of outcomes that are conducive (or otherwise) to the best lives. This is an empirical endeavor that I am neither equipped nor interested in answering in this paper, although certainly morally relevant upshots may arise from an investigation into the matter [13].

Within the present parameters, then, I argue that antinatalism supports the PPB in a way that sidesteps the main objections laid out against the PPB. Insofar as we are interested in reducing the amount of harm in the world, as antinatalism calls us to, we may begin to see the supplantation of such objections as alternative reasonable accounts of the good life and, most importantly, the nonidentity problem. I begin by first explicating the PPB and the objections against the PPB. I then review the kinds of pressures I refer to when contemplating what antinatalism tells us about prospective parenthood. I then consider how antinatalistic considerations weigh up against the objections that are laid out against PPB.

## 1 Principle of Procreative Beneficence and Its Objectors

The PPB posits affirmative moral obligations on prospective parents to birth a child (among a set of possible children) with the most desirable set of genes. Formally

*PPB*: couples (or single reproducers) should select the child, of the possible children they could have, who is expected to have the best life, or at least as good a life as the others, based on the relevant, available information [1]. (p. 415)

It is noteworthy that Savulescu now holds this as a *pro tanto* duty, where the obligation that arises from PPB ought to be understood as a moral reason which must be weighed against other moral reasons to determine what we most have reason to do. In this sense, the moral obligation that arises from PPB is a defeasible one if there are other, stronger reasons to act contrary to this obligation [6].^1^

Acclaimed authors have written extensively in opposition to the PPB, primarily questioning either the underlying metaphysical or normative assumptions that the PPB implicitly endorses. Alan Holland [6], for example, points out that the PPB relies on an underlying conception of what makes a good life. According to Holland's objection, since the conception of the good life that Savulescu implicitly accepts in crafting his PPB, we ought also to pay attention to alternative conceptions of the good life. Unfortunately for the PPB, these alternative conceptions afford the PPB fewer means to achieve operationality. Where Savulescu operationalizes the ‘best life’ as the life with the most well‐being [1], Holland points to such alternative accounts as a life of religious devotion, a Stoic account of the good life, and the view that a worthwhile life may be the best life [6, pp. 491–492]. Holland posits that if we take these alternative views seriously, which, he argues, we might do with as much strength as we view a well‐being maximization view, then there are at least these three ways of contemplating the good life through which the PPB is paralyzed. Simply put, the view that the best life is one that is committed to religious devotion imposes no such requirement that well‐being is maximized. The same goes for a Stoic view of the best life, which posits that the good life was that which made you least vulnerable to the vicissitudes of life via cultivating the virtues [6, p. 492]. Again, no such requirement for prospective parents to maximize the well‐being of their future children arises. On the contrary, it seems even more plausible that a Stoic would find praiseworthy the individual who was born with a genetic disadvantage but otherwise cultivated their virtues to the extent that they were well insulated from the struggles of life. Finally, the view that a worthwhile life is the best life tells us that being born with impediments does not preclude an individual from worthwhile lives, so that even if a prospective parent might reasonably wish that their children be born without serious impediments, a child who is otherwise born with such impediments might still live a worthwhile – or *the best* – life.

The final view that Holland points to is the view from which many objectors to the PPB derive their arguments. Indeed, the most vocal objector to the PPB, Peter Herissone‐Kelly, does this. He points out that the PPB fails to adequately distinguish between an external‐sense and an internal‐sense of betterness [14]. An external perspective of betterness attends to betterness *simpliciter*, in an agent‐independent manner, while an internal perspective of betterness attends to a specific agent for whom something is better. The PPB implicitly accepts an external sense of betterness, Herissone‐Kelly argues, but leaves much to be desired when one contemplates the internal sense of betterness. *For whom*, if anyone, will the PPB benefit? Herrisone‐Kelly invites us to consider two prospective embryos that may be implanted: A (a better‐life embryo) and B (a worse‐life embryo). The PPB would obviously impose a moral obligation (assuming that the obligation is not defeated) for prospective parents to implant embryo A, and not embryo B. However, an *internal‐sense* understanding of betterness makes the PPB inoperable. This is because if parents had decided to implant embryo B instead, the person that embryo B becomes might have a good life worth living. If B's parents had, counterfactually, opted to respond to the PPB and implanted A instead, then B would not have had their life worth living. From this distinction, Herissone‐Kelly argues in a later book chapter that the sorts of considerations that inform the PPB are silenced in the sense that a consideration that operates as a reason in one context is rendered inoperative in another [4].

This objection is heavily reminiscent of Parfit's nonidentity problem. According to Benjamin Meir Jacobs [15], the debate on the ethics of reproductive decision‐making in a post‐genetic age is heavily dependent on acceptable solutions to this problem. Inasmuch as that which is bad intuitively must be bad *for someone* [19], it is as odd that we consider implanting embryo A instead of B for the purpose of benefitting A and avoiding harming B. While intuitively we might accept that it is at least morally permissible to implant embryo A instead of B, the nonidentity problem explicates why this may not be the case. If, suppose, embryo A is implanted and embryo B is not, then the person that embryo B would have grown into would not exist. How, then, might we claim that causing embryo B to exist is a harm if there is no legitimate target of harm? Much like Herrisone‐Kelly's objection above, the nonidentity problem calls to mind the possibility that although embryo B might suffer from some condition that makes it the worse‐life embryo compared to A, the person that embryo B gives rise to could, still, enjoy a life worth living despite its genetically poorer start in life. To make this clear, consider two possible worlds:

W_1_: Embryo A is implanted, and embryo B is not implanted.

W_2_: Embryo B is implanted, and embryo A is not implanted.

Although I have stipulated that embryo A is the better‐life embryo, it is entirely possible that embryo B, in W_2_, can enjoy a life worth living. Choosing W_1_ instead of W_2_, while obviously better for embryo A, is not necessarily better for embryo B. So long as embryo B has a life minimally worth living, then the nonidentity problem makes it so that choosing W_2_ is not *bad*, because all things considered, embryo B benefits from being brought into the world despite the harm that it encounters in coming into existence. This, therefore, comes to contest the PPB directly. At least, it severely threatens the normative foothold it has on our intuitions—how are we to endorse a principle that wants us to be sensitive to the goods and bads of coming into existence when we might have reasons to reconsider the metaphysical legitimacy of its targets?

## 2 Antinatalistic Pressures and the Weighty Obligations View

At this juncture, I should first lay out the foundations of my defense of the PPB. It should come as no surprise that parents owe moral and legal duties of care to their progeny [17]. It is worthwhile to note that antinatalists, although they might not explicate it, seem to endorse what Brandt [18] refers to as the *Weighty Obligations View*. This view posits that “procreative obligations arise as a consequence of the general duty to compensate those we harm” (p. 788). Since procreation begets harm to the prospective child, we can be said to owe weighty obligations to our prospective children. As it happens, antinatalism tells us plenty about the kinds of risks and harms we impose upon our children via procreation. We can begin from the observation that harm avoidance is, at the risk of overgeneralization, a universal moral prerogative. It is here that most arguments for antinatalism arise.

In examining the plausibility of wrongful life suits, where children take legal action against their parents for causing them to exist in a miserable life, Seana Shiffrin [19] argues, much along the lines of the weighty obligations view, that parents may be liable for causing children to exist, because

Even though procreators may benefit their progeny by creating them, they also impose substantial burdens on them. By being caused to exist as persons, children are forced to assume moral agency, to face various demanding and sometimes wrenching moral questions, and to discharge taxing moral duties. They must endure the fairly substantial amount of pain, suffering, difficulty, significant disappointment, distress, and significant loss that occur within the typical life. (pp. 136–137)

Although Shiffrin is not expressly an antinatalist, her upshot from her legal analysis is that if burdens and risks of further harms are to be imposed upon someone to bestow upon them some benefit without that person's consent, it should be done with voluntary assumption of responsibility (p. 146). For this reason, Shiffrin is commonly cited as an antinatalist figure [20]. Consent, here, becomes an operative of normative interest to antinatalists (and, in extension, how antinatalism views the duties that parents owe their children). This is because children cannot possibly consent to coming into existence, and in extension, cannot consent to being harmed. It seems that we have, as Harrison [21] puts it, a *prima facie* duty not to subject others to lives without first gaining their consent. While some objectors might appeal to hypothetical consent, the mileage this objection sees remains suspect [22].

But this essay attends to what we owe to our children on the assumption that they will come into existence. By this point, this much should be obvious: antinatalism points out that because children (1) are seriously harmed or subject to significant risk of harm vis‐à‐vis existence, and (2) cannot consent to existing, we should owe weighty moral obligations to them in reparation for bringing them into existence. It appears to me an intuitive extension of the antinatalist thesis that although abstinence from procreation is preferable, if a child is created, then the least we could do is to ensure that the harm that we impose upon the child is adequately taken care of, broadly construed.

## 3 Antinatalism's Defense of the PPB

First and perhaps most obvious is how antinatalism directly contradicts Holland's and Herrisone‐Kelly's objection from an internal‐sense understanding of good. Whereas PPB objectors invite us to consider how an embryo without the best set of genes can give rise to a life perfectly worth living, antinatalism implicitly calls for an external‐sense understanding of the good.

An obvious defense of the external‐sense understanding of good arises from some principle of agent neutrality, which might posit that we ought to consider goodness on some global scale. “Everybody to count for one, nobody for more than one,” as Bentham writes [23]. However, this defense is piecemeal because it begs the question. That is, it already assumes that net good in the world, however defined, counts more than phenomenal, or lived‐experience, good. Vacuously, the external‐sense is right because the external‐sense is right.

I think that antinatalism makes a valuable contribution here. Because of a normative focus on harms, antinatalism can offer a valuable response to the internal‐sense argument. Here, instead of attending to the possibility of a life worth living from a set of suboptimal (in a PPB sense^2^) genes, antinatalism asks us to consider the extent to which the person suffers in their life. This consideration, together with the view of weighty obligations, gives rise to a normative priority to ensure the least amount of suffering in a prospective life. The important, yet perhaps nonobvious, conceptual shift here is that antinatalism is agnostic to whether a child has a life worth living (except, perhaps, Benatar's antinatalism). So, a prospective child arising from a set of suboptimal genes may well have a life worth living in the future—this is much compatible with antinatalism. Instead, the position demands that we should bring to life that child who would have the lowest propensity for internal‐sense suffering. A corollary version of the PPB can thus be explicated from antinatalism:

*Antinatalism‐Corollary PPB*: couples (or single reproducers) should select the child, of the possible children they could have, who is expected to *experience the least amount of suffering in life*—*or the lowest risk of significant suffering in life*—among the set of embryos—based on the relevant, available information.

This is surely a small conceptual shift from the original PPB. However, if we take the pressures of antinatalism and its adjacent assertions seriously, then the corollary version shifts our attention toward the likes of Shiffrin's and Harrison's arguments from consent, married with the weighty obligations view. Prospective people do not consent to being born, the narrative might go, so we ought to ensure that, against the baseline (assumed) neutrality of non‐existence, prospective people are genetically best equipped to be kept safe from harm. Against Herrisone‐Kelly's thought experiment between embryo A (the better‐life embryo) and embryo B (the worse‐life embryo), then, it matters little—as it relates to the debate about the PPB— to the antinatalist if B will proceed to have a life worth living. Embryo B cannot lament failing to be brought into the world if it were not implanted. However, it can reasonably lament the suffering that it encounters in the future if it were implanted, even if it evaluates its life as worth living *in toto*. Inasmuch as we employ a comparative metaphysical view of harm [24, 29], our moral prerogative shifts away from whether, all things considered, embryo B gives rise to a life worth living, and instead to the amount of harm we can reasonably assess embryo B to undergo in its lifespan against embryo A. If this is reasonable, then we can see how an antinatalist PPB might posit moral reasons to choose the implantation of embryo A over embryo B.

Next, the PPB and its defense famously require interaction with Parfitian nonidentity problems as stated above, but responding to antinatalist pressures can help us consider PPB in a manner that sidesteps the need for a lengthy detour into the metaphysics of person‐affecting normative claims. This is because antinatalism, broadly construed, is responsive to the amount of harm and suffering that arises in the world. As a theory, antinatalism cares not about who experiences the harms of coming into existence, but that existence implies harm, *simpliciter*. In considering antinatalist ethics, then, we see reasons to consider the amount of net harm in the world—that is, reasons to prefer an external‐sense understanding of good instead of an internal‐sense understanding of good.

But, at the risk of speaking (or writing) past Herissone‐Kelly's work, I should posit an internal‐sense argument for how non‐existence might be better for B than it is for A. Indeed, antinatalism might endorse a positive argument that even if the genetically suboptimal embryo proceeds to have a life worth living, this fails to imply the case that it has a life worth starting.

Between two embryos, A (the better‐life) embryo and B (the worse‐life) embryo, we can instead opt to consider the extent to which the embryos have lives worth starting. In Benatar's defense of antinatalism against the nonidentity problem, he points out that there is a significant morally relevant difference between lives worth starting and lives worth continuing. The former points to future‐life cases, while the latter points to present‐life cases. Benatar points out, here, that a common error is to evaluate both future‐ and present‐life cases with the same kind of normative standards as we do for present‐life cases. Instead, he points out that “the judgment that an impairment is so bad that it makes life not worth continuing is usually made at a much higher threshold than the judgment that an impairment is sufficiently bad to make life not worth beginning” [11, p. 23]. I think Benatar is right, here, that there is an asymmetry with which we should judge the extent to which lives are worthwhile to continue, or to start. For example, it is far easier to justify opting against choosing to implant an embryo that we know will grow into a person who suffers tremendously from Huntington's Disease than it is to claim that a person with the same affliction ought not carry on existing. This points to the central intuition that the threshold of harms to justify preventing a birth is far lower than a similar threshold that justifies terminating a life.

On risk, Matti Häyry, another icon in antinatalist philosophy, also points out that a needs‐based axiology deepens this distinction [25]. As existing people, we take risks as a matter of necessity in our daily lives because we *need* to. For example, we undertake risks every day by crossing the street or flying in aircraft because modern lives place such needs upon us. However, prospective people have no such needs. Benatar and Häyry, through different arguments, point to a distinction between lives worth starting and lives worth continuing.

But what does this distinction tell us in relation to the PPB and sidestepping the nonidentity problem? Simply put, I think that it downplays the consideration of the genetically suboptimal child having a life worth living despite its suboptimal genetic beginnings. It could, surely, be the case that the embryo becomes somebody with a life worth living. However, moving away from stipulations, we cannot reasonably *know* this until the person exists, and sufficient life data is available for such an evaluative claim. At the chronological position of implantation, all we have are genetic odds. Häyry's position on risk, together with Benatar's distinction between the normative thresholds of when lives are worth starting or continuing, can inform our position about the PPB. Between the two embryos, then, the antinatalist would be interested to ask: which of these lives are worth starting, where the life worth most worth starting between the two is the life that is most likely to have the lowest amount of overall harm, and the lowest risk of a life that is not worth living? As mentioned, framing procreative priorities as such sidesteps the nonidentity problem by downplaying a critical feature of the problem. Specifically, it sidesteps the possibility that any prospective child might grow into a person with a life worth living, since the possibility of a life worth living is no longer a concern. At least, this consideration enjoys a far reduced normative force.

Savulescu comes quite close to endorsing a similar view. In his defense of the PPB and in response to Michael Parker's [13] criticisms, Savulescu [5] comes close to what I hope to argue in this paper, although not quite to the same effect. Firstly, Savulescu points out that to a large extent, we intuitively know what makes a life better or worse. These evaluations comprise a surprisingly large proportion of our interactions with each other. We set priorities in health, research, social services, and the distribution of limited resources as a function of evaluating lives (p. 284). Medical services, for example, exist because we acknowledge that being ill is worse than being healthy, and we ought to attend to remaining healthy as much as possible. Secondly, Savulescu also points out that acting in response to PPB is an attempt to improve the odds of doing well in an uncertain world of difficulty, threat, and misfortune (p. 284), so that we want to equip our future children with the best odds at success as we can. These points are important in antinatalism, and indeed, Savulescu is not far from an antinatalist conclusion. Antinatalism, broadly construed, takes no issue with the notion that some lives are better than others. Antinatalism is also extremely sensitive to the risk of coming into the world and the harms that accrue to a child when they are born [26, 27]. The primary difference between Savulescu's intuition and that of an antinatalist, then, might be the threshold at which risk is worth taking. But this is not the focus of this study.

So, I hope that I have made clear my argument: the sensitivity to harm and risk that accompanies a transition from non‐existence into existence that pervades antinatalism is valuable in informing the debate about the legitimacy of the PPB. Specifically, I think that antinatalism supports the PPB. If we are to have children at all—as antinatalists are happy to admit that we will, despite their arguments [29]—then we should want to reduce the kinds of suffering that antinatalists raise to, at least, attempt to alleviate the suffering that comes with procreation. Teo [27], for example, points out that as a matter of practical necessity, existence comes with harms. But, on the contrary, the requisite features of a life worth living do not obtain with the same kinds of epistemic certainty. He now (verbally) refers to this observation as the Likelihood Asymmetry, in defense of his Moderate Antinatalism. Insofar as the PPB can tip the scales for a prospective child so that the harms thrust upon them via existence may be outweighed to address the Likelihood Asymmetry, we can begin to see the benefit of endorsing the PPB. An important observation should be noted here. Antinatalism is a pessimistic philosophy that frequently accepts a negative‐utilitarian‐type axiology,^3^ where harm weighs more heavily on our normative reasons‐generation than benefit. So, then, antinatalist philosophy generally asks that we be particularly sensitive to harms. Perhaps this weakens my case against the intuitions of those who reject such an axiological position, but I leave the theoretical normative work for other, more interested, and more qualified colleagues to discuss.

So, insofar as an action‐guiding principle serves to reduce harm in the world, within reason,^4^ antinatalism (or antinatalistic pressures) supports the PPB. This might look like the following:

1. Where reasonably practicable, if we are to procreate, we ought not to expose our prospective children to more harm from coming into existence than is necessary (Antinatalism‐Corollary PPB). Further, prospective parents have an obligation to reduce, as far as is reasonably practicable, the harms and risks of harms that come with coming into existence (Weighty Obligations View). Call this the *Antinatalistic Pressures Premise*.
2. Comparing between possible worlds W_1_ and W_2_, where both worlds are equal except that W_1_ contains less overall harm than W_2_, W_1_ is choiceworthy. Call this the *Harms Avoidance Premise*.
3. In response to (1), choosing to implant an embryo among a set of embryos that does not have the best set of genes (call this embryo the genetically‐suboptimal embryo) is to expose that embryo—and subsequently the person that that embryo ultimately develops into—to unnecessary risks of harm. I call these risks of harm unnecessary because there is at least one embryo among the set of embryos that would not be exposed to the same risks as the genetically suboptimal embryo. Insofar as the genetic suboptimality of the embryo is concerned, any risk of harm that the embryo is subsequently exposed to is, therefore, unnecessary (or, non‐necessary).
4. In response to (2), if a prospective parent responds to the moral obligation from PPB and subsequently implants a genetically‐optimal embryo A in W_1_ and the genetically‐suboptimal embryo B in W_2_, we have reasons to believe that W_1_ would contain less overall harm than W_2_ in an impersonal sense. Therefore, W_1_ is preferable to W_2_.
5. Because of (3) and (4), the PPB is conducive to the moral prerogative to reduce harms where harms (and risks of harms) are unnecessary, within reason. Insofar as this moral prerogative is legitimate, we have reasons to believe that the PPB is valuably action‐guiding.

This seems like a trivial observation, but I think not. The counterarguments that have been mounted on the PPB, as listed above, have appealed to myriad normative arguments, but seem to focus on who the principle benefits, and not necessarily who is harmed.

Antinatalism and its moral prerogative to avoid harms, therefore, responds well to the counterarguments from Holland and Herissone‐Kelly above. Holland's three alternative views (being a religious perspective of the best life, a Stoic account of the best life, and a worthwhile‐life view of the best life) are all compatible with a prerogative to avoid harms. Both the religious and Stoic perspectives may consider harm as ultimately beneficial, or as a kind of struggle that assigns more meaning to a life if harms are overcome. However, we should not conflate these perspectives to say that harm is not *bad*. The religious and Stoic perspectives might develop some reason to think about harms that may be *outweighed* by, or *instrumental* to, some greater good (e.g., a choiceworthy afterlife, or the cultivation of virtues). However, I doubt that if asked about harm to no such benefit, these perspectives would posit harm as inherently *good*.

## 4 Conclusion

In this short essay, I have explicated a case for how developments in antinatalism contribute to the debate on the legitimacy of the Principle of Procreative Beneficence. In doing so, I defend the PPB against criticisms mounted by such authors as Holland and Herissone‐Kelly. I argue that taking antinatalist normative pressures seriously, even if one does not subscribe to an antinatalist position, the PPB is conducive to helping us navigate our moral obligations to our prospective children. Specifically, since antinatalism accentuates our weighty moral obligation not to unnecessarily harm our prospective children, the PPB is a valuable principle to guide actions in alignment with antinatalist pressures.

## Data Availability

Data sharing is not applicable to this article as no data sets were generated or analyzed during the current study.

## Endnotes

1 Savulescu, 2014, as cited in Herissone‐Kelly [4].

2 N.B.: Henceforth, every mention of “suboptimal genes,” or similar, points to a PPB sense of optimum. I want to make this clear because I want to discuss gene sets only within the confines of PPB and PPB‐adjacent literature. This is important to highlight to separate the subject matter at hand from the historical, horrific eugenics that arise from bigotry that, needless to say, I condemn.

3 For example, see Benatar [11]; Häyry [34].

4 I include this quantifier to avoid straw‐man arguments against negative utilitarianism. Without this quantifier, an interlocutor might argue that if negative utilitarianism is true, then we shouldn't accept as morally acceptable some case in which benefits drastically outweigh harms. E.g., that a negative utilitarian is committed to condemn plucking a hair off a person's head if it means the spontaneous cessation of global poverty. This is not a fair assessment of negative utilitarianism in its modern form, but I have neither the space nor intention to defend negative utilitarianism in this paper. As it relates to antinatalism, see Häyry [32].

## References

[1] J. Savulescu , “Procreative Beneficence: Why We Should Select the Best Children,” Bioethics 15, no. 5–6 (2001): 413–426, 10.1111/1467-8519.00251. 12058767

[2] L. Gantsho , “The Principle of Procreative Beneficence and Its Implications for Genetic Engineering,” Theoretical Medicine and Bioethics 43, no. 5–6 (2022): 307–328, 10.1007/s11017-022-09585-0. 35882747

[3] R. Ranisch , “Procreative Beneficence and Genome Editing,” American Journal of Bioethics 22, no. 9 (2022): 20–22, 10.1080/15265161.2022.2105435. 36040888

[4] P. Herissone‐Kelly , “The Lack of an Obligation to Select the Best Child: Silencing the Principle of Procreative Beneficence.” in Parental Responsibility in the Context of Neuroscience and Genetics, vol. 69, eds. K. Hens , D. Cutas and D. Horstkötter (Springer International Publishing, 2017), 153–166, 10.1007/978-3-319-42834-5_10.

[5] J. Savulescu , “In Defence of Procreative Beneficence,” Journal of Medical Ethics 33 (2007): 284–288, 10.1136/jme.2006.018184. 17470506 PMC2598126

[6] A. Holland , “The Case Against the Case for Procreative Beneficence (PB),” Bioethics 30, no. 7 (2016): 490–499, 10.1111/bioe.12253. 27000681

[7] B. Saunders , “Procreative Beneficence, Intelligence, and the Optimization Problem,” Journal of Medicine and Philosophy 40, no. 6 (2015): 653–668, 10.1093/jmp/jhv026.. 26453079

[8] T. Douglas and K. Devolder , “Procreative Altruism: Beyond Individualism in Reproductive Selection,” Journal of Medicine and Philosophy 38, no. 4 (2013): 400–419, 10.1093/jmp/jht022. 23856478 PMC3905710

[9] L. Doyal , “Is Human Existence Worth Its Consequent Harm?,” Journal of Medical Ethics 33, no. 10 (2007): 573–576, 10.1136/jme.2006.020156. 17906053 PMC2652792

[10] D. Benatar and D. Wasserman , Debating Procreation: Is It Wrong to Reproduce? (Oxford University Press, 2015). https://academic.oup.com/book/25806?login=true.

[11] D. Benatar , Better Never to Have Been: The Harm of Coming Into Existence (Clarendon Press; Oxford University Press, 2006).

[12] C. Li , “When My Grandfather Stole Persimmons Reflections on Confucian Filial Love,” Dao 7, no. 2 (2008): 135–139, 10.1007/s11712-008-9059-8.

[13] M. Parker , “The Best Possible Child,” Journal of Medical Ethics 33, no. 5 (2007): 279–283, 10.1136/jme.2006.018176. 17470505 PMC2598117

[14] P. Herissone‐Kelly , “Procreative Beneficence and the Prospective Parent,” Journal of Medical Ethics 32, no. 3 (2006): 166–169, 10.1136/jme.2005.012369. 16507665 PMC2564476

[15] B. M. Jacobs , “Is There a Moral Obligation to Select Healthy Children?,” Journal of Medical Ethics 41, no. 8 (2015): 696–700, 10.1136/medethics-2014-102400. 25370604

[16] M. A. Roberts , “The Nonidentity Problem,” in *The Stanford Encyclopedia of Philosophy* (2024), https://plato.stanford.edu/archives/fall2024/entries/nonidentity-problem/.

[17] P. Marcus , “Parental Responsibilities: Reformulating the Paradigm for Parent–Child Relationships Part 2: Who Has Responsibilities to Children and What Are These Responsibilities?,” Journal of Child Custody 14, no. 2–3 (2017): 106–133, 10.1080/15379418.2017.1370407.

[18] R. Brandt , “Procreative Obligations and the Directed Duty of Care,” Journal of Applied Philosophy 41, no. 5 (2024): 785–803, 10.1111/japp.12755.

[19] S. V. Shiffrin , “Wrongful Life, Procreative Responsibility, and the Significance of Harm,” Legal Theory 5, no. 02 (1999): 117–148, 10.1017/S1352325299052015.

[20] M. Häyry and A. Sukenick , “Imposing a Lifestyle: A New Argument for Antinatalism,” Cambridge Quarterly of Healthcare Ethics 27 (2023): 1–22, 10.1017/S0963180123000385. 37496143

[21] G. Harrison , “Antinatalism, Asymmetry, and an Ethic of *Prima Facie* Duties,” South African Journal of Philosophy 31, no. 1 (2012): 94–103, 10.1080/02580136.2012.10751770.

[22] A. Singh , “The Hypothetical Consent Objection to Anti‐Natalism,” Ethical Theory and Moral Practice 21, no. 5 (2018): 1135–1150, 10.1007/s10677-018-9952-0.

[23] M. E. L. Guidi , “‘Everybody to Count for One, Nobody for More Than One’: The Principle of Equal Consideration of Interests From Bentham to Pigou,” Revue d’études Benthamiennes no. 4 (2008), 10.4000/etudes-benthamiennes.182.

[24] E. Pitcovski , “Explaining Harm,” Philosophical Studies 180, no. 2 (2023): 509–527, 10.1007/s11098-022-01909-z.

[25] M. Häyry , “Procreative Generosity: Why We Should Not Have Children,” Philosophies 8, no. 5 (2023): 96, 10.3390/philosophies8050096.

[26] T. L. T. Marcus , “Best to Possibly Not Be: A Prudential Argument for Antinatalism,” Bioethics 38, no. 8 (2024): 722–727, 10.1111/bioe.13330. 38923008

[27] M. Hӓyry , “A Rational Cure for Prereproductive Stress Syndrome,” Journal of Medical Ethics 30, no. 4 (2004): 377–378, 10.1136/jme.2003.004424. 15289525 PMC1733883

[28] M. Häyry , “If You Must Make Babies, Then at Least Make the Best Babies You Can,” Human Fertility 7, no. 2 (2004): 105–112, 10.1080/14647270410001699063. 15223759

[29] D. Purves , “Harming as Making Worse Off,” Philosophical Studies 176, no. 10 (2019): 2629–2656, 10.1007/s11098-018-1144-1.

[30] M. Häyry , “If You Must Give Them a Gift Then Give Them the Gift of Nonexistence,” Cambridge Quarterly of Healthcare Ethics 13 (December 2022): 1–12, 10.1017/S0963180122000317. 36511114

[31] M. Häyry , “Exit Duty Generator,” Cambridge Quarterly of Healthcare Ethics 33, no. 2 (April 2024): 217–231, 10.1017/S096318012300004X. 36799026

